# Improving Physicians' Capacity for Chronic Obstructive Pulmonary Disease Care through Blended E-learning: A Pilot Study in Bangladesh

**DOI:** 10.7759/cureus.3808

**Published:** 2018-12-31

**Authors:** Md. Nazim Uzzaman, Shakila Banu, GM Monsur Habib, AHM Enayet Hossain, Muhammad Jahangir Kabir, Md. Rizwanul Karim, Mohammod Rafiqul Islam, Mohammad Habibur Rahman Sarker, Md. Jasim Uddin, Aftab Uddin

**Affiliations:** 1 Epidemiology and Public Health, International Centre for Diarrhoeal Disease Research, Dhaka, BGD; 2 Internal Medicine, Bangladesh Primary Care Respiratory Society, Dhaka, USA; 3 Ophthalmology, Directorate General of Health Services, Dhaka, BGD; 4 Family Medicine, Bangladesh Primary Care Respiratory Society, Feni, BGD; 5 Epidemiology and Public Health, Directorate General of Health Services, Dhaka, BGD; 6 Miscellaneous, International Center for Diarrhoeal Disease Research, Dhaka, BGD

**Keywords:** blended learning, e-learning, copd, training, spirometry

## Abstract

Patients with chronic obstructive pulmonary disease (COPD) are often under diagnosed and managed without evidence-based approach in primary care settings. This may be due to gaps in knowledge and practice of using updated COPD guidelines by the physicians in public and private sectors in Bangladesh. To our knowledge, this is the first study in Bangladesh which aims to evaluate a blended e-learning approach for building capacity of physicians working at low-resource environments on COPD patient care.

In total, 32 practicing physicians were enrolled where 16 received training via blended approach and 16 received training via traditional classroom-based approach. Using a standard examination procedure and assessment approach both groups were assessed and results were documented.

No statistically significant differences were found in the scores of theory (knowledge) and in the total scores (theory plus practicum) of both groups indicating that learning objectives were achieved in both the groups though the scores were significantly higher in practicum of the traditional learning group. Besides, Likert-scale-based self-reported pre-post changes indicate that both the groups were confident (statistically significant) in the management of COPD. Most importantly, the blended group had a minimal disruption of their services as they attended face-to-face only during the practicum sessions.

Overall, the blended e-learning appears to be a feasible approach of training for physicians on standard management of COPD especially in health human resource-poor settings in Bangladesh.

## Introduction

Chronic obstructive pulmonary disease (COPD) is currently the fourth leading cause of death [1] which induces substantial economic and social burden in the world [2]. Globally, the COPD burden is anticipated to increase in the coming decades because of continued exposure to its risk factors and aging of the population [3]. In Bangladesh, around 6.5 million people over 40 years of age suffer from this disease [4]. The prevalence of COPD was estimated at 13.5% by the Global Initiative for Chronic Obstructive Lung Disease (GOLD) criteria and 10.3% by the lower limit of normal (LLN) criteria in Bangladesh [5].

In most patients, COPD is associated with co-morbidities and is generally under diagnosed and insufficiently managed in primary care [6]. There is a considerable gap between current COPD guidelines and what is actually done at general practice by the primary care physicians [7-8]. On the other hand, due to human resources for health (HRH) crisis and inequitable distribution, there is a huge shortage of qualified physicians in the rural and semi-urban areas of Bangladesh [9]. As a result, despite having learning needs, it often becomes difficult for a primary care physician to attend various training courses via traditional classroom-based approach leaving their practices. In blended e-learning approach, the use of digital technology and other non-traditional methods are integrated to add flexible, ‘anytime, anywhere’ learning, adaptable to work pressure and personal conditions [10-11]. It provides the opportunity to personalize learning through repeated exercise of specific and complex parts of the content as often as desired until they are profoundly understood and can be applied in practice [12].

A few studies have empirically examined the effectiveness of blended learning in medicine and reported an increase in trainee satisfaction with the content, better use of time in class, increase in knowledge and promote self-directed learning [13-14]. To our knowledge, this is the first study in Bangladesh which aims to evaluate a blended e-learning approach for building capacity of physicians working at low-resource environments on COPD patient care.

## Materials and methods

Study design

It was a pilot study on blended versus traditional learning of COPD for physicians working at primary care settings. A total of 32 physicians were enrolled in the pilot project where 16 physicians received training via bended approach (e-learning + face-to-face) and 16 physicians received training via traditional classroom-based approach (only face-to-face). The study was conducted from March 14, 2018 to June 28, 2018.

Study participants

The study participants were physicians working at Upazila health complexes (UHCs) of two purposively selected districts in Bangladesh. The participants for the blended e-learning were from the UHCs of Manikganj district and the participants for traditional classroom-based training were from the UHCs of Kishoreganj district of Bangladesh. The training for traditional learning group was conducted at the International Centre for Diarrhoeal Disease Research, Bangladesh (icddr,b) where as the face-to-face training for blended e-learning group was conducted at Manikganj district to further minimize their service disruption.

The Government of Bangladesh (GOB) provides healthcare services to its rural people through health facilities called UHCs. It provides inpatient and outpatient care, primary healthcare, family-planning services, and other preventive healthcare services and represents 31% of the government health sector [15-16].

Blended e-learning

The blended e-learning method integrates (i) current classroom activities of 16 hours for practical in two days and (ii) e-learning of 24 hours for theory in three weeks to allow participants self-paced convenient learning. The practical part was conducted via classroom-based face-to-face training. The e-learning was provided through specific resources delivered via distant learning management system of icddr,b. Furthermore, Facebook group was used as a learning tool for group discussion and problem solving [17].

Traditional classroom-based learning

The total training hours and contents for theory (24 hours) and practical (16 hours) component were same as the blended learning; however, it was delivered solely via classroom-based face-to-face approach. Total training hours were distributed over five days.

Learning contents

The major learning contents were patho-physiology of COPD, risk factors, critical issues in history‐taking, interpretation of spirometry tracing, treatment options, care planning and self‐management for the patients.

Evaluation

Pre-post Assessment of Confidence Level

Immediately before starting and after the end of training, a pretest and a post-test were taken from the participants using the same questionnaire to assess their confidence changes in terms of knowledge and skills using 5 rating Likert scale, with 1 representing ‘strongly disagreed’; 2 ‘disagreed’; 3 ‘neither agreed nor disagreed’; 4 ‘agreed’; 5 ‘strongly agreed’.

Final Examination

A final examination was conducted at the end of training where both knowledge and skills of participants were assessed. This examination consisted of written and oral component including clinical skill test. Written examination was of 30 marks with 30 multiple choice question (MCQ) and oral examination was of 10 marks on spirometry tracing interpretation.

Feedback from Participants

At the end of training, participants were given a structured form to evaluate the course. Furthermore, they were asked about problems or challenges they faced to go through the e-learning module and requested to give feedbacks for further improvement of the training.

Analysis

To assess for normality of the data, descriptive statistics were computed. The analyses of the data proceeded using paired sample t-test for the pre-post knowledge and skill data. The examination scores of both groups were assessed using two-sample t-test with equal variances. Missing data were excluded from the analysis using pairwise and listwise deletion. Statistical significance was assumed at P value <0.05. Stata Statistical Software 2013 (StataCorp LP, College Station, Texas, USA) was used for data analysis.

Ethical statement

The study was approved by the Ethical Review Committee of icddr,b.

## Results

The participants of blended e-learning group and traditional learning group had an average experience of eight years and 10 years of patient care respectively. In both the groups, male participants were more than the female participants (Table 1).

**Table 1:** Baseline characteristics of participants n: Number of participants; COPD: Chronic obstructive pulmonary disease.

Variable	Traditional learning	Blended e-learning
Age (year)		
Median	35	36.5
Interquartile range	6	5.5
Sex - n (%)		
Male	11 (73)	10 (62)
Feamle	4 (27)	6 (38)
Duration of practice (year)		
Median	8	10
Interquartile range	5	5
Current position		
Medical Officer	9 (60)	14 (88)
Residential Medical Officer	1 (7)	1 (6)
Consultant	4 (26)	1 (6)
Upazila Health Officer	1 (7)	
Previous training on COPD - n (%)		
Yes	0	0
No	15 (100)	16 (100)

The final examination results revealed that the mean scores for theory, practical and total were 18.81 (±2.37), 8.06 (±0.44) and 26.88 (±2.25) for traditional learning group and, 18.47 (±1.55), 7.2 (±0.68) and 25.67 (±1.63) for blended e-learning group respectively. Although the scores in practical examination was found significantly higher (P value 0.0002) among the participants of traditional learning group, there were no statistically significant differences found in theory (P value 0.6372) and total scores (P value 0.0993) between these groups (Table 2).

**Table 2:** Comparison of examination scores SD: Standard deviation.

Variable	Traditional learning group			Blended e-learning group			P value
	Mean	SD	% of score	Mean	SD	% of score	
Theory score	18.81	2.37	62.7	18.47	1.55	61.6	0.6372
Practical score	8.06	0.44	80.6	7.2	0.68	72	0.0002
Total score	26.88	2.25	67.2	25.67	1.63	64.18	0.0993

Self reported pre-post confidence changes of participants were found significant for both groups (Table 3).

**Table 3:** Comparison of self-reported confidence changes of participants COPD: Chronic obstructive pulmonary disease; SD: Standard deviation.

Area of knowledge and skill	Traditional learning group			Blended e-learning group		
	Pre-test	Post-test	P value	Pre-test	Post-test	P value
	Mean (SD)	Mean (SD)		Mean (SD)	Mean (SD)	
Understand the causes and patho-physiology of COPD	3.44 (0.96)	4.68 (0.47)	0.0001	2.71 (0.91)	4.57 (0.51)	0.0001
Take comprehensive respiratory history from a patient	3.37 (1.14)	4.5 (0.51)	0.0005	3.57 (0.93)	4.57 (0.51)	0.0072
Obtain accurate objective measurements from a patient	2.81 (0.83)	4.43 (.051)	0.0001	2.5 (1.01)	4.35 (0.63)	0.0001
Identify abnormal spirometry patterns	2.31 (1.01)	4.37 (0.5)	0.0001	1.85 (0.86)	4.42 (0.51)	0.0001
Diagnose COPD patient properly	3.12 (1.08)	4.31 (0.47)	0.0004	2.64 (0.92)	4.57 (0.51)	0.0001
Understand the various treatment strategies for COPD	3.06 (1.06)	4.5 (0.51)	0.0001	2.71 (0.99)	4.5 (0.51)	0.0001
Teach patients the correct way to use a range of inhalers	3.43 (0.96)	4.68 (0.47)	0.0002	3.14 (1.16)	4.71 (0.46)	0.0007
Use clinical guidelines to choose appropriate medications	2.68 (1.07)	4.25 (0.57)	0.0001	2.57 (0.85)	4.28 (0.46)	0.0001
Manage acute exacerbation of COPD properly	3.56 (1.03)	4.5 (0.51)	0.0063	2.85 (0.94)	4.35 (0.49)	0.0002
Know pulmonary rehabilitation	2.5 (0.96)	4.12 (0.61)	0.0001	2.21 (0.89)	4 (0.78)	0.0001
Tailor medication schedule to the patient’s daily routine	2.68 (0.94)	4.12 (0.61)	0.0003	2.64 (0.92)	4.21 (0.57)	0.0001
Review long-term therapeutic plan	2.93 (1.12)	4.06 (0.68)	0.002	2.57 (0.85)	4 (0.55)	0.0002
Give specific information to worries expressed by the patient	3.31 (0.94)	2.45 (0.44)	0.0019	2.42 (0.75)	4.28 (0.46)	0.0001

In terms of course evaluation using 5 rating Likert scale, all participants of both groups rated that the course objectives were met and overall the course was successful (Table 4).

**Table 4:** Course evaluation by participants n: Number of participants.

Area of evaluation	Traditional learning group - n (%)					Blended e-learning group - n (%)				
	Strongly disagreed	Disagreed	Neither agreed nor disagreed	Agreed	Strongly agreed	Strongly disagreed	Disagreed	Neither agreed nor disagreed	Agreed	Strongly agreed
The course contents were relevant	0	0	0	9 (56)	7 (44)	0	0	0	4 (27)	11 (73)
Theoretical sessions were effective	0	0	1 (6)	9 (56)	6 (38)	0	0	0	8 (53)	7 (47)
Practical sessions were effective	0	0	0	3 (19)	13 (81)	0	0	0	4 (27)	11 (73)
Training materials were adequate	0	0	2 (13)	4 (25)	10 (62)	0	0	0	5 (33)	10 (67)
Facilities for training were adequate	0	0	1 (6)	5 (31)	10 (63)	0	0	0	7 (47)	8 (53)
The objectives of the course were met	0	0	0	5 (31)	11 (69)	0	0	0	6 (40)	9 (60)
Overall the course was successful	0	0	0	8 (50)	8 (50)	0	0	0	6 (40)	10 (60)

## Discussion

This study generates a novel finding that blended e-learning is an effective approach to build the capacity of primary care physicians on COPD in Bangladesh. Although blended e-learning was found as an effective approach to training in other medical fields [18-19], to our best knowledge, its effectiveness on COPD was not explored before this study. Our study found no statistically significant differences when compared the theory scores between the groups, even though, the theoretical contents were delivered via e-learning in a blended approach and via face-to-face in traditional classroom-based approach. Despite utilizing the same approach (face-to-face) for both groups in practical contents, the participants of the traditional learning group did better as compared to the participants of the blended group. The factors which might contribute to the low score in this group are insufficient facilities in the training venue such as inadequate space, fixed seats, frequent load shedding as well as unavailability of some training logistics during training. However, the comparison of the total score between the groups reveals no statistically significant differences.

Some studies have also found that the blended approach is as effective as face-to-face learning [20-21]. In our study, self-reported pre-post confidence changes of participants on essential knowledge and skills for management of COPD were found significant for both groups. Cook et al. [22] reported no difference between objective and subjective assessments in knowledge scores; however, differences in personality traits of learners might influence the changes in confidence level, as people with greater confidence tend to give higher ratings on subjective assessments than people who are less confident [23]. In our study, all participants of blended group thought that the approach was better than the traditional approach. We found similar responses about blended e-learning that it allowed participants to learn on convenient time and review online materials as often as necessary [24]. Nonetheless, the opinions of participants towards blended e-learning are found very positive; still there are many challenges to address including internet connectivity and offline accessibility. Smyth et al. also reported that some participants felt the online component was invasive in their daily life, specifically following a day at work [25].

In regard to the weakness of our study, the small sample size was a concern. This blended e-learning was successfully implemented in a small group of physicians; however, the feasibility of implementing this approach in a large group remains uncertain. There was no scope to randomize the participants into the learning groups. The skills or competencies of participants were not measured in a real patient-based setting. Furthermore, self-report by physicians may not always correspond to reality.

## Conclusions

In conclusion, blended learning can be a feasible alternative to traditional classroom-based learning to deliver training on specific health topics including COPD for physicians in Bangladesh. It promotes greater flexibility of self-paced learning with some extra advantages especially in reducing the interruptions of daily activities in patient care settings. Further research is required to validate the outcomes and investigate whether similar findings are apparent in a large group of physicians.
